# Recommendations for promoting user agency in the design of speech neuroprostheses

**DOI:** 10.3389/fnhum.2023.1298129

**Published:** 2023-10-18

**Authors:** Narayan Sankaran, David Moses, Winston Chiong, Edward F. Chang

**Affiliations:** ^1^Kavli Center for Ethics, Science and the Public, University of California, Berkeley, Berkeley, CA, United States; ^2^Department of Neurological Surgery, University of California, San Francisco, San Francisco, CA, United States; ^3^Weill Institute for Neuroscience, University of California, San Francisco, San Francisco, CA, United States; ^4^Memory and Aging Center, Department of Neurology, University of California, San Francisco, San Francisco, CA, United States

**Keywords:** BCI, speech-decoding, neuroethics, neurotechnologies, neuroprosthesis

## Abstract

Brain-computer interfaces (BCI) that directly decode speech from brain activity aim to restore communication in people with paralysis who cannot speak. Despite recent advances, neural inference of speech remains imperfect, limiting the ability for speech BCIs to enable experiences such as fluent conversation that promote *agency* – that is, the ability for users to author and transmit messages enacting their intentions. Here, we make recommendations for promoting agency based on existing and emerging strategies in neural engineering. The focus is on achieving fast, accurate, and reliable performance while ensuring volitional control over when a decoder is engaged, what exactly is decoded, and how messages are expressed. Additionally, alongside neuroscientific progress within controlled experimental settings, we argue that a parallel line of research must consider how to translate experimental successes into real-world environments. While such research will ultimately require input from prospective users, here we identify and describe design choices inspired by human-factors work conducted in existing fields of assistive technology, which address practical issues likely to emerge in future real-world speech BCI applications.

## Introduction

A speech brain-computer interface (BCI) that directly translates brain activity into speech has the potential to improve the quality of life and autonomy of people with paralysis who cannot speak (Branco et al., 2021; Vansteensel et al., 2022). However, the extent to which these benefits are realized in future applications will rest on how successfully a device promotes *agency* – that is, working through a speech BCI, users must have the ability to author and transmit messages that enact their intentions on the world (Goering et al., 2021).

What might the properties of such an agency-enabling device be? Relative to traditional BCIs that indirectly infer speech through visual cursor control or letter-by-letter using evoked response paradigms (e.g., P300 spellers), a BCI that directly decodes speech from cortical activity would be efficient, and grant users more immediate access to the rich expressive capabilities of language. Indeed, evidence suggests that people with speech paralysis often prefer strategies that directly decode their speech attempts (Branco et al., 2021) and most desire to hold normal conversation, prioritizing qualities such as speed, reliability, and ease-of-use in an assistive-communication device (Huggins et al., 2011; Nijboer et al., 2014; Peters et al., 2015). Thus, our conception of a BCI that optimally promotes agency is one which generates speech outputs at a conversational rate by precisely and reliably decoding the dimensions of brain activity that represent information with communicative intent.

Unfortunately, achieving this ideal is nontrivial because neural inference of speech is currently imperfect. Our scientific understanding of both healthy and impaired language production remains incomplete, and current neural interfaces impose limits on the spatiotemporal resolution and signal-to-noise ratio of data, restricting our ability to observe linguistic processes in the brain that would enable robust real-time decoding (Shen et al., 2023). As a result, even the most successful speech BCIs to-date have not matched the speed and accuracy of natural spoken language (Metzger et al., 2023; Willett et al., 2023). Furthermore, the vast majority of existing research has been conducted within highly controlled laboratory settings, using artificial paradigms that decode predefined speech targets from known temporal epochs of activity. Ensuring that BCIs maintain the qualities necessary for promoting agency in real-world contexts will pose an even greater challenge. Given that neural inference is imperfect, trade-offs and design choices must be guided by human factors to ensure robust function as speech BCIs transition from the laboratory to less controlled environments.

To address these challenges, we consider the neurobiological, linguistic, engineering, and human factors upon which the agency-enabling capacities of a speech BCI depend, and we provide recommendations to guide their development moving forward. We restrict our focus to clinical BCI applications aimed at restoring speech-based communication, and primarily consider intracranial recording modalities due to their demonstrated ability to facilitate real-time speech decoding from brain activity.

### Ensuring volitional control over when speech is decoded, what information is decoded, and how it gets expressed

In order to precisely and reliably broadcast information, speech BCIs must first know *when* users intend to speak. Within those temporal windows, BCIs must know *what* signals to decode, translating only the dimensions of neural activity associated with communicative information. Finally, BCIs must know *how* users wish to convey this information and allow for the flexible expression of speech output. Below, we describe existing and emerging strategies that move toward these goals.

A speech BCI must detect the general temporal windows during which users intend to speak. To achieve this, recent BCI systems include a separate speech-detection module that functions prior to decoding speech content in order to identify the temporal onset and offset boundaries of intended speech (Kanas et al., 2014; Moses et al., 2019; Dash et al., 2020; Moses et al., 2021). Rather than continuously engaging a decoder, we argue that such an approach is both efficient and can safeguard mental privacy. While erroneously detected speech events would degrade usability (e.g., by causing accidental exits from standby mode), studies have shown that highly accurate speech detection is attainable in real-time intracranial BCI systems. For example, (Kanas et al., 2014) achieved 92% accuracy using time-frequency representations of electrocorticographic (ECoG) data to classify voice-related activity. More recently, (Moses et al., 2021) used an artificial neural network to segment continuous ECoG data into probable speech events, resulting in the successful detection of 98% of word attempts. Thus, inclusion of a separate speech-detection module represents a feasible first step toward providing users with volitional control over BCI outputs.

Aside from the detection of speech-related windows, decoders must selectively read dimensions of activity corresponding to information that the user wishes to externalize. A critical consideration here is the specific level of the language-production hierarchy associated with the neural signals being decoded. It is well established that spoken language results from a series of hierarchically-organized neural processing steps (Hickok, 2012). An idea originates within a conceptual-semantic system, before moving through word-level (lemma), phonological, and articulatory processing stages that progressively transform abstract ideas into precise speech acts. In theory, BCIs may generate speech outputs by decoding activity corresponding to any stage within this linguistic hierarchy. For instance, recent fMRI work decoded meaningful language from high-level semantic representations that reside across a highly distributed network of brain regions (Tang et al., 2023). In contrast, the majority of speech-BCI research has focused on decoding low-level articulatory and phonological signals within sensorimotor and auditory regions of the cortex (Martin et al., 2014; Herff et al., 2015; Ramsey et al., 2018; Anumanchipalli et al., 2019; Moses et al., 2021; Proix et al., 2022). The impact of these decoding strategies on user-agency become clear when we consider the extent to which the brain implements various levels of the linguistic hierarchy during behaviors that lack certain qualities – such as physical articulation or communicative intent – that characterize healthy spoken language.

In order to elaborate on these considerations, we first propose a standard terminology in reference to various speech-related behaviors. Healthy speakers produce what we refer to as *overt speech*. By contrast, in order to convey messages, BCI systems may require users with paralysis to engage with speech-decoders in two different ways. First, users may engage in *attempted speech*, involving a deliberate attempt to articulate. To the extent that residual control of the vocal tract muscles is retained, this may result in vocalization or silent (“mimed”) articulation. Second, users may engage without articulating, typically referred to in the literature as *imagined speech.* Importantly, despite the absence of articulation, imagined speech is by definition a volitional act with communicative intent. In contrast, we refer to all private processes – ones that lack both articulation and communicative intent – as *internal speech.* While terminology in reference to these behaviors has been inconsistent across the speech-decoding literature, we stress the need for standardized nomenclature to facilitate collective empirical inquiry into their neural basis.

While overt speech, by definition, recruits representations across the language-production hierarchy (from conceptual-semantic to articulatory), the extent to which different representations are encoded during attempted, imagined, or internal speech is less clear. For the purpose of conferring agency, speech decoders must target representations that meet two distinct requirements. On one hand, to effectively restore speech to people with paralysis, including to those who are fully locked-in, decoders must target representations that remain sufficiently active even during attempted or imagined speech (when articulation is severely limited or absent). On the other hand, to safeguard mental privacy, decoders must target representations that *only* remain active during attempted or imagined speech (when speaking is intended) and not during internal speech. The former requirement thus demands *sensitivity* to communicative dimensions of brain activity, while the latter requirement demands *specificity* to only those dimensions.

Let us first address the former requirement. Recently, researchers decoded meaningful sentences from articulatory representations during attempted speech in individuals with severe speech paralysis (Moses et al., 2021; Metzger et al., 2023). This demonstrates that – even in cases where articulatory control is heavily impaired – the brain continues to implement low-level speech-motor programs during deliberately attempted speech. A compelling further possibility is that – even in fully locked-in patients – deliberate speech attempts elicit articulatory representations that closely resemble those of healthy overt speech. Future work is needed to determine whether this is indeed the case. In contrast with attempted speech, it is likely that imagined speech recruits a distinct cortical pathway that largely circumvents speech-motor regions. Indeed, evidence from early imaging studies (Murphy et al., 1997; Shuster and Lemieux, 2005) as well as recent electrophysiological recordings (Proix et al., 2022) suggest that articulatory representations are only weakly encoded during imagined speech. While this may be a particularly salient point for people with damage to speech-motor cortex, it remains unknown whether articulatory organization in these individuals returns as they gradually learn to use a speech BCI through adaptation and frequent usage, potentially in a rehabilitative manner. Additionally, alternate strategies that decode imagined speech from phonological (Brumberg et al., 2016) or somatosensory (Wandelt et al., 2022) representations have shown promise. In particular, a large body of research has shown that speech perception and imagery activate overlapping representations within auditory cortex (see Martin et al., (2018) for a review), suggesting that decoders trained on activity elicited during listening can be used to decode imagined speech (Martin et al., 2014). In sum, for the purpose of restoring speech to those with paralysis, low-level articulatory and phonological brain signals are a promising target for decoders.

Next, let us consider the requirement that decoders respect mental privacy. While the neural representation of internal speech remains poorly understood, we argue that such private thoughts are unlikely to be encoded within low-level regions associated with motor or sensory processing. As already discussed, articulatory signals are weakly encoded in the absence of a deliberate speech-motor goal (Proix et al., 2022). Given that these articulatory programs are not robustly implemented during (volitional) imagined speech, they are highly unlikely to be implemented during (private) internal speech. Nonetheless, it is critical for future work to compare the anatomical and functional organization of imagined versus internal speech in order to isolate the neural basis of communicative intent – a topic which has received no empirical neuroscientific attention to our knowledge. This issue is of growing importance, particularly in light of the fact that it is possible to decode speech from high-level semantic representations measured noninvasively (Huth et al., 2016; Tang et al., 2023). The *amodal* nature of semantic brain signals allows for decoders that are highly domain-general – perhaps trained to decode during communicative acts but capable of reading speech during altogether non-communicative contexts (e.g., while watching a silent movie). Research must focus on whether and how semantic activity differentiates contexts in which intention to communicate is present versus absent.

Finally, the communication modality through which speech BCIs ultimately convey output has significant implications for agency and ownership of decoded messages. While text-based communication is an important modality for interfacing with digital technology, synthesis of speech-sounds directly from cortical activity could offer more naturalistic and expressive forms of communication that include linguistic dimensions, such as intonational prosody and syllabic stress patterns, in addition to merely the identity of speech tokens. Traditionally, an approach that incorporates these other dimensions has been challenging in people with paralysis because – in the absence of overtly produced output – the precise temporal alignment between neural activity and intended speech remains unknown. However, modern machine-learning methods that utilize temporal convolutions, data augmentation, and connectionist temporal classification (Moses et al., 2021; Metzger et al., 2022, 2023; Willett et al., 2023), alleviate this issue by enabling training and inference without precise alignment. These approaches thus offer potential to drastically improve the expressive capabilities of speech BCIs.

While the factors discussed so far are critical for providing volitional control, achieving a high-performance speech neuroprosthesis will ultimately require improvements in the neural inference of speech. Such improvements could be realized by better neural interface design, more advanced computational methods, and progress in our neuroscientific understanding of speech production. Intriguingly, improvements may also be realized by introducing knowledge outside of users’ brain activity into the inference process, via the incorporation of language models.

### Agential implications of incorporating language models into decoding pipelines

Linguistic sequences have statistical structure, such that the probability of possible future outputs are modulated by what came before. Language models (LMs), which have internalized this statistical structure by training on independent large language corpora, are being incorporated into real-time BCI pipelines to aid neural decoding. Currently, the operation of LMs within speech BCIs is analogous to an “autocorrect” function, seeking to improve neural inference either directly at the neural-decoding stage (Sun et al., 2020), retrospectively by interpreting decoded word probabilities such that final output sentences conform with highly probable sequences (Moses et al., 2021), or proactively by first generating a set of candidate continuations and then evaluating the most likely candidate conditioned on the neural activity that eventually occurred (Tang et al., 2023).

In essence, these approaches introduce knowledge outside of users’ brain activity into the inference process, potentially morphing a person’s intended message to conform with the statistical trends found within language corpora. Because of this, concerns have been raised that the incorporation of LMs into decoding pipelines may improve efficiency at the expense of user control (Maslen and Rainey, 2021). While we agree with the principles motivating this concern, we highlight the need to consider use of LMs in the context of current speech-decoding capabilities. Given that neural inference of speech is inaccurate, the use of LMs serves to drastically improve the accuracy of decoded outputs (Moses et al., 2021; Metzger et al., 2022). That is, relative to unaided neural decoding, the incorporation of LMs aligns decoded outputs more closely with users’ intended expression. So long as LMs provide such accuracy gains, the promotion of user agency will not necessarily involve constraining their use.

While we have considered the use of LMs as an “autocorrect” function within BCI pipelines, in principle, they may also be used to predictively generate output, analogous to an “autocomplete” function. Indeed, given that speed is a priority for prospective BCI users, some suggest that until speech can be decoded at fluent conversational rates, “developers should explore rate enhancement features… such as word and phrase prediction” (Peters et al., 2015). If LMs are used in such a way, it will be necessary to decouple speed from accuracy, and consider the trade-off between efficiency and user control proposed by prior literature (Maslen and Rainey, 2021). The question of where a given speech BCI should sit along the speed-accuracy continuum is for individual users to decide, and must therefore be made customizable and context-dependent in the design of BCIs. This is particularly necessary if certain “high-stakes” contexts (e.g., legal testimonies) require confidence in the veracity of decoded output, even if this comes at the expense of speed (Chandler et al., 2021). Flexibility along the speed-accuracy continuum is just one of many requirements that must be met if speech BCIs are to successfully transition from experimental to real-world environments – a topic that we turn to next.

### Bringing speech BCIs into real-world environments

As speech BCIs continue to advance in research settings, it is important to keep the primary clinical goal of this technology in focus: to improve autonomy and quality of life for patients who cannot speak. Therefore, in parallel with the continued scientific pursuit of fast, precise, and reliable speech BCIs, research must consider how to translate existing experimental successes into real-world settings in a manner that ultimately realizes those patient benefits.

To effectively achieve this transition, we argue that BCI developers should implement human-centered design (HCD) frameworks, soliciting input from those with acute needs and engaging with them in an iterative process of co-design (Boy, 2017). To our knowledge, no HCD work has been conducted specifically for speech BCIs, and we strongly advocate for such future research. Additionally, BCI developers may consider design insights from existing assistive technologies (Hill et al., 2021). Specifically, commercial augmentative and alternative communication (AAC) devices already help many patients with physical disabilities to communicate (Beukelman et al., 2007; Linse et al., 2018). These devices are designed to be user friendly and robust, and implement important features that facilitate agency, including the ability to personalize the voice of generated speech outputs and perform error-correction before finalizing outputs. Speech-language pathologists (SLPs) work closely with patients to customize AAC devices, tailoring decisions to each patient’s needs and capabilities. In turn, this improves adoption and reduces abandonment of AAC devices (Johnson et al., 2006). While these insights are not intended to replace the crucial user-perspectives that HCD research on speech BCIs would provide, they may nonetheless prove useful for practical implementations of speech BCIs in the future. In this section, we highlight three design elements that may promote agency in future real-world BCI applications: (1) maximally reliable control outputs, (2) error-correction capacity, and (3) communication customization.

Despite recent advances in speech BCI performance within research settings (Metzger et al., 2023; Willett et al., 2023), decoding is unlikely to reach the accuracy of spoken language in the near future. Therefore, speech BCI systems will need guardrails against generation of unintended outputs. One solution involves using maximally reliable and highly detectable signals as control signals for high-level command over the system. This is analogous to fail-safe mechanisms and multimodal-input options that are common in commercial AAC devices (Hurtig et al., 2019). A control signal could comprise any attempted command that reliably generates a salient signal. Although attempted speech can be used for this purpose, a non-speech motor attempt may be preferable because its neural signature is known to be distinct from attempted speech (Penfield and Boldrey, 1937). Indeed, researchers have recently shown that attempted hand movements can be reliably decoded from the same brain implant that decodes speech while generating signals that are highly distinguishable from attempted speech (Metzger et al., 2022, 2023). Other approaches may leverage residual physical capabilities instead of neural signals (e.g., eye gaze, foot movements, or unintelligible vocalizations). The identification of these control outputs should be tailored to – and developed in collaboration with – individual users. This mechanism may then be used to access application-control menus, engage error-correction modes (see below), indicate the endorsement of messages in high-stakes scenarios (Chandler et al., 2021) and mitigate the overall risk of users losing control over BCI outputs in the event of inaccurate decoding. Additionally, as current speech BCIs rely on intracranial neural interfaces that cannot be physically switched on or off, this control mechanism could also be used to toggle the streaming of neural data or speech-decoding function on or off as desired.

Given that decoded speech is more error-prone than spoken language, there will likely be a need for error-correction capabilities, which is a common feature in traditional AAC devices (Thompson et al., 2013). Many possible strategies exist for enabling error correction in speech BCIs. Again, these strategies must be developed and refined with input from prospective users. Possible strategies include: (1) Suggesting high-likelihood alternatives using the decoded probabilities from a classifier or beam-search algorithm, (2) Using attempted spelling or writing to correct individual letters (Willett et al., 2021; Metzger et al., 2022), and (3) Allowing the repeat of certain words and/or the entire utterance to aggregate probabilities across the multiple attempts for a refined prediction. Engagement of an error-correction process can be initiated by using the highly reliable signal discussed in the previous paragraph. For BCI systems that convey decoded messages as speech-sounds with low-latency, error-correction may prove more difficult, as decoded outputs may be generated contemporaneously with speech attempts, leaving no time for post-hoc correction. Such a case may require generation of an additional phrase to correct what was previously output.

Finally, customization over the communication parameters is an important consideration for promoting agency. In traditional AAC devices, users can customize various features of the communication interface, including keyboard layouts, the presence of autocomplete options, the vocabulary of the system, and the voice of the generated speech (Moorcroft et al., 2019). Speech BCI users may benefit from similar customizations, including: (1) How adaptive they want the language model to be to their long-term communication patterns; (2) The relative weight that the language model carries during decoding; (3) Common high-utility phrases for reliable access; and (4) The voice of the speech synthesizer and, potentially, the face of a digital avatar to accompany synthesized speech output and further embody the user, both of which can be personalized (Metzger et al., 2023). Users may wish to customize these features independently or in consultation with SLPs, caregivers, and BCI support technicians.

## Discussion

In summary, clinically relevant speech BCIs must afford volitional control over when and what is communicated through the device, and how exactly this is expressed. Language-modeling techniques are currently necessary to reach usable performance levels with existing approaches, but the rate of advancement of speech BCIs in recent years illustrates the need to consider how reliance on language models can affect agency. Moving forward, speech BCI research must look beyond the science and toward the ultimate needs of prospective users situated in everyday environments. We advocate for HCD approaches to achieve this. However, in this article we have considered how insights from other domains of assistive technology could address practical issues that speech BCI users are likely to face in real-world contexts.

While we have largely focused on software implementations, advances in hardware will also continue to improve the agency-promoting qualities of speech BCIs. Advances in neural interface design will yield better signal resolution (for increased performance), data transmission (for wireless data telemetry to enable fully implantable devices, reducing infection risks and daily setup effort), and portability (Weiss et al., 2019). In addition, in an increasingly digital world, the optional integration of speech BCIs with users’ personal devices (e.g., smartphones, laptops) would improve their autonomy, and provide expanded opportunities for social interaction, education, and employment (Zickler et al., 2009).

## Data availability statement

The original contributions presented in the study are included in the article/supplementary material, further inquiries can be directed to the corresponding author.

## Author contributions

NS: Conceptualization, Formal analysis, Investigation, Writing – original draft. DM: Conceptualization, Formal analysis, Investigation, Writing – review & editing. WC: Supervision, Writing – review & editing. EC: Supervision, Writing – review & editing.
